# Animal Tissue Mineralization: An Overview of Disease Processes, Comparative Pathology, and Diagnostic Approaches

**DOI:** 10.3390/biom16010096

**Published:** 2026-01-07

**Authors:** Eliana De Luca, Fabio Del Piero

**Affiliations:** 1 College of Veterinary Medicine, Louisiana State University, Baton Rouge, LA 70803, USA; edeluca@lsu.edu; 2 LSU Diagnostics-Louisiana Animal Disease Diagnostic Laboratory, Department of Pathobiological Sciences, School of Veterinary Medicine, Louisiana State University, Baton Rouge, LA 70803, USA

**Keywords:** mineralization, calcification, comparative pathology, disease, infection-associated calcification

## Abstract

Calcium deposition within soft tissues is a significant pathological process, bearing significant implications for animal and human health. It is classified into four categories, including dystrophic, metastatic, idiopathic, and iatrogenic. It involves multiple molecular mechanisms. Vascular calcification includes medial artery mineralization, siderocalcinosis in equine cerebral arteries, and vitamin D-induced arterial mineralization in multiple species. Renal and urinary mineralization occurs with kidney disease, uremic gastropathy, and ethylene glycol toxicity. Calcinosis cutis is associated with renal insufficiency and systemic fungal infections and is commonly observed in dogs with hyperadrenocorticism, while calcinosis circumscripta occurs at pressure points secondarily to trauma. Multiple pathogens are responsible for soft tissue calcification; they can be zoonotic and include *Mycobacterium* spp., *Brucella* spp., *Toxoplasma gondii*, and *Echinococcus granulosus*, underscoring the translational role of veterinary medicine surveillance from a public health standpoint. In addition, the placental chorioallantois is frequently affected by idiopathic or infection-induced calcification, highlighting the convergence of metabolic dysregulation and infectious mechanisms. Tissue calcifications provide valuable insights into disease mechanisms and diagnostic challenges, with comparative pathology serving as a powerful tool to enhance our understanding of these processes from a One Health standpoint.

## 1. Introduction

Comparative pathology is a field that compares diseases in animals and humans to gain insight into both human and animal health. It involves studying disease processes across numerous different species, using animal models to understand human diseases, and exploring the translational potential of these models. The depositaries of this science are the board-certified veterinary pathologists that evaluate many cases of natural diseases daily via necropsies and histopathology, as well as ancillary tests including microbiology and molecular techniques.

Pathological mineralization consists of the deposition of insoluble and inorganic minerals in soft tissues. In humans and animals, including those of zootechnical interest, the most important mineralizing process is calcification, a phenomenon invariably associated with the morphogenesis and remodeling of hard tissues, such as bone and teeth, under physiological conditions and occasionally involving soft tissues in pathological conditions. This phenomenon constitutes a significant and diagnostically relevant finding in comparative pathology, as ectopic calcium deposition may disrupt normal tissue architecture, exacerbate local inflammatory response, and represent an indication of an underlying pathological state driven by metabolic imbalance, cellular injury, or chronic inflammation [1,2]. The pathogenesis of soft tissue calcification encompasses several mechanisms that range from passive physicochemical precipitation in necrotic or degenerative tissue to complex, cell-mediated processes. Macroscopically affected tissues may form white to yellowish, firm, gritty, chalky plaques of variable size, while histologically, calcium deposits generally appear as basophilic with a granular to crystalline morphology on hematoxylin and eosin (H&E) staining [2,3]. Special histochemical stains are routinely employed to confirm the presence and nature of calcium salts, such as von Kossa and Alizarin Red S stains [4]. However, a definitive interpretation requires integration of microscopic findings with clinical history and biochemical data, such as serum calcium and phosphorus levels, as well as species-specific physiological reference intervals and mineralization patterns.

The aim of this review is to provide a comprehensive summary of pathological calcification in comparative pathology, focusing on classification, underlying cellular and molecular mechanisms, diagnostics, organ- and species-specific patterns, and current research directions. Emphasis is placed on integrating histopathological findings with clinical and systemic data as well as on understanding the underlying molecular mechanisms, in order to gain a deeper understanding of this complex pathological process from a veterinary pathology standpoint.

## 2. Classification and Mechanisms of Calcification in Animals

The classification of pathologic tissue calcification into four main categories, including dystrophic, metastatic, idiopathic, and iatrogenic calcification, is based on the distinct pathophysiological mechanisms as well as clinical and pathological findings [3]. The most prevalent form of ectopic calcium deposition is dystrophic calcification, which occurs in injured or necrotic tissues because of localized cellular damage, inflammation, degeneration, or thrombosis [3,5]. This process is distinguished by the deposition of primarily calcium phosphate despite normal serum concentrations of calcium and phosphate [5,6]. In this scenario, calcium binds to membrane phospholipids, lipid-derived byproducts, and denatured proteins that accumulate at sites of cell death, creating nucleation foci for mineral crystallization leading to a passive and localized mineralization process [7]. Dystrophic calcification is frequently identified across various organ systems, such as in myocardial and skeletal muscle fibers in the context of vitamin E or selenium deficiency, which is a medical condition commonly referred to as nutritional myopathy, enzootic myopathy, or “white muscle disease” that is well described in young ruminants and foals [5]. Similarly, calcinosis cutis is classified as a poorly understood form of epithelial and collagenous calcification mainly described in canine endogenous or iatrogenic hyperglucocorticoidism and calcinosis circumscripta. The latter represents a more localized deposit of calcium salts that is attributed to repetitive trauma and tissue necrosis [8]. Furthermore, dystrophic calcification may arise in the setting of localized connective tissue disorders, panniculitis, neoplasms, chronic infections, trauma, and thermal injury [9]. In contrast to dystrophic calcification, metastatic calcification arises secondary to systemic imbalance of calcium and phosphate levels, such as hypercalcemia and hyperphosphatemia or a combination of both [3,10]. Tissues commonly affected by metastatic calcification include the intima and tunica media of vessels, especially in the lungs, pleura, endocardium, kidneys, and stomach, followed by renal tubular and pulmonary alveolar basement membranes, myocardium, and gastric mucosa. In veterinary species, frequent etiologies include chronic kidney disease, vitamin D intoxication, primary or secondary hyperparathyroidism, and paraneoplastic syndromes [3]. Furthermore, metastatic calcification has been reported in dogs affected by fungal diseases, such as systemic blastomycosis or paecilomycosis [11,12,13,14]. Idiopathic mineralization occurs without tissue damage or abnormality in the metabolism of calcium or phosphorus. In small animals, idiopathic calcinosis occurs more frequently than dystrophic or metastatic calcinosis [15]. Calcium is commonly deposited in the soft tissues of the tongue and subcutis of a dog’s paw pads as amorphous nodular aggregates without identifiable causative factors [3]. Iatrogenic calcification represents a distinct pathological entity characterized by the deposition of calcium salts due to direct exposure to exogenous calcium-containing compounds. This typically occurs following topical or parenteral administration of calcium-rich solutions, which induce localized tissue injury and subsequent mineralization within the damaged tissues [5]. Furthermore, metaplastic calcification, also known as heterotopic ossification, consists of the pathological formation of mature bone or cartilaginous tissue within chronic lesions of soft tissue calcification. Notable examples in pathology include ectopic bone formation within paravertebral muscles [16], osseous metaplasia in dogs affected by salivary mucocele [17], fibrodysplasia ossificans affecting the cervical soft tissues described in a German Shepherd dog [18], and development of mature lamellar bone within the aortic valve in a horse [19].

The deposition of calcium salts in soft tissues is a complex process involving multiple cellular and molecular mechanisms [20]. Mitochondria play a central role in the regulation of intracellular calcium homeostasis and serve as primary sites of calcium sequestration during pathological calcium overload. This increased and dysregulated uptake contributes to mitochondrial dysfunction while promoting favorable conditions for subsequent calcification [3]. Dystrophic calcification arises in the context of local tissue injury, which is driven by the release of phosphate-binding proteins from necrotic cells in response to tissue damage, inflammation, or hypoxic conditions [9]. On the other hand, metastatic calcification has primarily been reported to be related to systemic calcium phosphate imbalances, rather than localized tissue injury. In this regard, elevated extracellular calcium concentrations can impair cellular regulatory mechanisms, leading to intracellular calcium accumulation and subsequent mineral deposition. This process is commonly associated with conditions characterized by persistent hypercalcemia, such as primary hyperparathyroidism, increased parathyroid hormone (PTH), or parathyroid hormone elated peptide (PTHrP) production in tumor cells, resulting in humoral hypercalcemia or pseudohyperparathyroidism [21]. In this regard, common examples in dogs include adenocarcinoma of the anal sac (≈90% of cases), lymphoma (≈20% of cases), and multiple myeloma (≈15% of cases) [3].

In physiological conditions, tissue mineralization results from an imbalance between osteogenic and inhibitory factors that regulate hydroxyapatite deposition in the extracellular matrix. Physiological mineralization is initiated within matrix vesicles that bud from hypertrophic chondrocytes, osteoblasts, and odontoblasts, where calcium and inorganic phosphate (Pi) crystallize as hydroxyapatite [22]. The ratio between Pi and inorganic pyrophosphate (PPi) is crucial as PPi inhibits crystal formation, whereas tissue-nonspecific alkaline phosphatase (TNAP) promotes mineralization by hydrolyzing PPi and generating Pi [22]. On the other hand, in a physiological context, nucleotide pyrophosphatase/phosphodiesterase-1 (NPP1) and the transmembrane protein progressive ankylosis homolog (ANKH) generate and transport PPi extracellularly, acting as calcification inhibitors [23].

Other osteogenic mediators include bone morphogenetic proteins (BMPs) that belong to the transforming growth factor beta (TGF-β) family and induce osteogenic differentiation by binding to heteromeric complexes of two types of Ser/Thr kinase receptors (BMP type I and type II receptors), leading to the activation of BMP target gene transcription [24]. More than 20 BMPs have been identified so far and BMP-2, -4, -6, -7, and -9 play a major role in bone morphogenesis [25].

Besides PPi, NPP1, and ANKH described previously, additional inhibitory factors include matrix Gla protein (MGP), which binds calcium phosphate nuclei and BMP-2, limiting calcification [26]. In addition, fetuin-A acts systemically as a mineral chaperone, forming soluble calciprotein particles that prevent ectopic mineral precipitation [26]. Osteopontin (OPN), a phosphorylated glycoprotein, binds crystal surfaces to inhibit hydroxyapatite growth and mediates macrophage-driven clearance of mineral aggregates [27]. Collectively, TNAP, BMPs, and matrix vesicles promote osteogenesis, while PPi, NPP1, ANKH, MGP, fetuin-A, and OPN act as inhibitory factors of calcification. Several studies reported that certain forms of calcification, including arterial calcification in horses, are triggered by regulated biomineralizing processes. Indeed, various proteins associated with bone mineralization were detected in calcified areas of the arterial walls, while VSMCs were demonstrated to be able to differentiate into an osteogenic and chondrogenic phenotype, leading to deposition of mineral within the blood vessels [4]. In addition, vascular calcification has been associated with compromised elastic compliance of the arterial wall, resulting in increased vascular stiffness, which represents a significant risk for organ dysfunctions [4]. Glucocorticoids, both endogenous and iatrogenic, play a pathogenic role in the context of calcinosis cutis. While the precise pathogenesis is still unclear, it has been demonstrated that glucocorticoids are able to downregulate calcification inhibitors and decrease mRNA expression levels of matrix Gla protein, osteopontin, and vascular calcification-associated factor (VCAF) [13]. In the saponification of necrotic adipose tissue, for example, in the peripancreatic adipose, there is a leakage of lipases which leads to the hydrolysis of free fatty acids that form insoluble calcium soaps, giving a chalky, off-white appearance to the injured tissues [3]. Collectively, the classification of calcification provides a framework for understanding the multiple manifestations in animals, highlighting the biological complexity of this process. Combining morphologic and molecular patterns is essential for an accurate diagnosis and pathogenesis understanding.

## 3. Diagnostic Approaches and Differential Diagnosis in Comparative Pathology

On H&E staining, calcium salts are typically described as basophilic, granular to amorphous deposits, which may be localized intracellularly, extracellularly, or in both compartments [9,13]. Although histological examination provides a presumptive characterization of minerals, the confirmation requires specific histochemical stains. Two classic calcium-specific stains routinely used in diagnostic pathology are von Kossa and Alizarin Red S [3]. In the von Kossa histochemical technique, metallic silver precipitates with calcium phosphate or calcium carbonate salts, resulting in the blackening of calcified tissue [3]. It is particularly useful in cases of chronic kidney disease, myofiber mineralization, metastatic calcification, and vascular mineralization [3]. On the other hand, Alizarin Red S binds directly to calcium ions, staining the mineral deposits bright orange to red. Unlike von Kossa, it reacts with calcium in necrotic and mineralized fibers, and it is commonly used to confirm early dystrophic mineralization and microcalcifications [9,13]. IHC represents a valuable tool for elucidating the molecular pathways involved in pathological mineralization [4]. Calcification of the media of peripheral arteries is known as Mönckeberg’s sclerosis (MS) in human medicine, and it is most commonly described in older and diabetic individuals. In a study conducted by Shanahan et al. [20], an upregulation of bone sialoprotein, alkaline phosphatase, bone Gla protein, and collagen II, all indicators of osteogenesis and chondrogenesis, was reported, further supporting the hypothesis that VSMCs undergo osteogenic differentiation [20]. Similarly, these osteogenic markers were also demonstrated in mineralizing vascular tissues in horses [4].

Regarding metastatic calcification, the evaluation of serum total and ionized calcium and phosphate, as well as parameters of renal functionality, are essential to reach a final diagnosis, as underlying etiologies commonly include chronic kidney disease, vitamin D toxicosis, primary hyperparathyroidism, and paraneoplastic syndromes [28].

Several endogenous and exogenous materials can resemble mineral deposits in histological sections, including hemosiderin and melanin, which may appear as granular, basophilic pigments and can be differentiated using Prussian blue and Fontana-Masson stains, respectively [3,29]. Exogenous or artefactual materials that may resemble minerals include formalin pigment, which appears as brown-black deposits and are von Kossa-negative [30].

An accurate diagnosis of pathological mineralization in veterinary medicine requires a comprehensive and integrated diagnostic approach, including animal clinical history and physical examination findings, complemented by serum measurements of calcium, phosphorus, and vitamin D. Several imaging techniques, including radiography and computed tomography, may aid in the detection and anatomical localization of mineral deposits, while histopathological examination remains the cornerstone for a definitive diagnosis complemented by von Kossa staining and immunohistochemical techniques.

Of interest, von Kossa silver staining specifically adapted to electron microscopy allowed the ultrastructural identification of calcium-binding sites in both bovine and porcine calcifying aortic valve interstitial cells (AVICs). Using such a procedure, acidic phospholipid-rich material resulting from cell membrane lysis and progressively layering at the edges of degenerating AVICs and their vesicular debris was found to play a major role in calcium nucleation [31]. Concerning immunohistochemical approaches, recent studies indicate that cytosolic phospholipase A2α (cPLA2α), an enzyme that hydrolyses membrane phospholipids, may play a role in the calcification of AVICs experimentally induced in vitro and in vivo, respectively. Under these conditions, increased cPLA2α expression was associated with progressive cytomembrane degeneration, with subsequent apatite crystal formation [32,33]. Indeed, a time-dependent increase in cPLA2α-immunopositive AVICs was paralleled by a progressive rise in AVIC positivity to von Kossa silver staining in vivo [33], whereas enzymatic inhibition in vitro results in a marked reduction in AVIC calcification [32].

## 4. Species-Specific and Organ-Specific Patterns of Calcification

Tissue calcification is associated with distinct morphological patterns and clinical implications that differ based on the organ system involved and the species affected. An overview of the calcification categories, with a description of organ- and species-related examples are reported in Table 1.

### 4.1. Vascular Calcification

Calcification of arteries represents a significant finding in multiple species. Arterial calcification is characterized by the deposition of calcium minerals within the tunica media, known as Mönckeberg’s sclerosis, or within the tunica intima, which is commonly associated with atherosclerotic lesions in humans [20]. In equine species, calcification of large arteries has been sporadically documented in racehorses at comparatively earlier ages than humans, where medial arterial calcification is mostly considered an age-related degenerative process [34]. In a study conducted by Arroyo et al. [4], a high prevalence (82%) of vascular calcification was found in Thoroughbreds and Standardbreds of approximately 4.4 years, primarily affecting the media of pulmonary artery branches, followed by the aortic and carotid trunks. On a gross examination, vascular calcification appears as a white to yellowish, firm plaque, with a gritty and brittle texture. The plaques can range from small, well-circumscribed areas measuring approximately 0.5 cm to multiple, extensive patches potentially involving the entire arterial wall. The vessels are commonly characterized by wall thickening, with lesions varying from mild with well-circumscribed foci of calcification between elastic fibers to extensive circumferential or transmural mineralization in the most severe cases [4,5]. Although the pathogenesis of medial artery calcification in horses is still controversial, growing evidence supports an association with intense athletic activity. Indeed, the condition has been described in 82% of Thoroughbred and Standardbred horses that died within two weeks of racing [34]. Lesions occur at a young age (2 to 5 years), often without a known metabolic or toxic insult, underscoring the clinical relevance of repeated hemodynamic stress during high performance that may contribute to vascular remodeling and mineralization. Indeed, contrary to humans, where an association with advancing age, diabetes, or renal diseases has been described [35], affected horses showed no evidence of systemic disease. In addition, equine systemic calcinosis is a degenerative and mineralizing myopathy that has occasionally been described in young adult Quarter horses and Paint horses showing lethargy, gait stiffness, and exercise intolerance but without an underlying etiology [36,37]. In the study conducted by Tan et al. [37], the main findings included dystrophic calcification of cardiac myofibers and renal tubules, as well as calcification of the tunica intima of submucosal intestinal vessels and focal hemorrhage in one horse.

On the other hand, siderocalcinosis is characterized by deposition of calcium and iron salts, and it is considered an incidental, age-related finding in the cerebral arteries of older horses [38]. Arterial medial calcification is also widely recognized in multiple animal species, including rabbits, aged guinea pigs, and rats, and etiologies include ingestion of calcinogenic plants (*Cestrum diurnum*, *Trisetum flavescens*, *Solanum malacoxylon*, and *Nierembergia* spp.), vitamin D toxicosis, renal insufficiency, and Johne’s disease in cattle caused by *Mycobacterium avium* subspecies *paratuberculosis* [3,5]. The affected large elastic arteries, particularly the aorta, may appear firm with raised white intimal plaques. Histologically, there is prominent basophilic granular mineral deposition within the elastic fibers or a complete circumferential ring of calcification within the medial layer [5].

Lastly, hemosiderotic plaques, also known as siderotic plaques, siderofibrotic plaques, siderocalcific plaques, siderotic nodules, or Gamna-Gandy (GG) bodies, are mostly common in dogs and are considered a sequela of prior trauma and hemorrhage. The term “siderotic nodule” refers to the granulomatous changes secondary to a hemorrhage caused by trauma, hypertension, neoplastic processes, or age-related changes. GG bodies refer to the so-called bamboo-shaped structures found within siderotic nodules, which consist of calcium and iron centered on connective tissue fibers and are morphologically similar to a typical fungal hyphae structure [39]. Siderotic nodules are commonly described within the fibrous connective tissue of the splenic capsule, trabeculae, and/or around the hilar vessels [5].

Vitamin D toxicosis is a well-recognized cause of arterial mineralization, predominantly affecting the aortic, coronary, and mesenteric arteries. Similarly, chronic renal vein thrombosis and uremic gastropathy are associated with metastatic calcification, particularly involving the arteries and arterioles of the gastric wall [40]. Interestingly, in cases of vitamin D toxicosis, calcification may also occur simultaneously in multiple soft tissues, affecting areas such as ligaments, tendons, pleura, pulmonary parenchyma, gastric mucosa, and vascular walls. Similarly, in the context of vitamin D intoxication and uremic gastropathy, the gastric mucosa and mucosal and submucosal vessels are both affected [3,5].

### 4.2. Renal and Urinary System Calcifications

**Figure 1:**
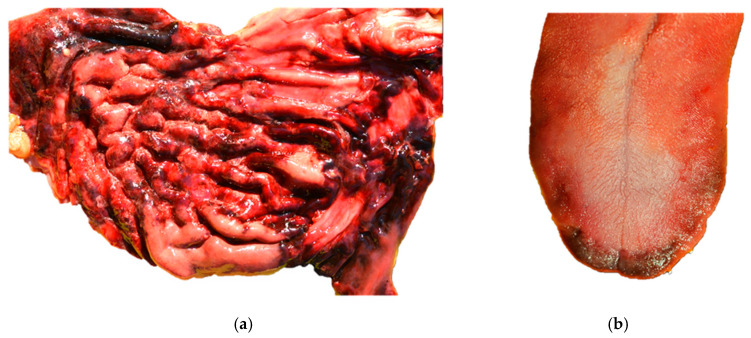
Soft tissue mineralization in domestic species. (**a**) Case L1219747, Dog, Maltese, 7 years. Stomach: mucosal necrosis, multifocal, subacute, and severe with hemorrhage and mineralization, accompanied by interstitial nephritis, lymphoplasmacytic, multifocal to coalescing, chronic, severe with extensive fibrosis, basement membrane mineralization, tubular degeneration, and glomerulosclerosis. Diagnosis: chronic end-stage kidney disease with secondary uremia; (**b**) case L1219747, Dog, Maltese, 7 years. Tongue: glossal necrosis, focally extensive, subacute, moderate. Diagnosis: chronic end-stage kidney disease with secondary uremia. Cutaneous soft tissue mineralization.

Calcification within the renal and urinary systems represents a frequent pathological finding in feline medicine, mostly associated with chronic kidney disease (CKD) and specific toxic insults [3]. CKD affects approximately 30% of cats older than 10 years [41], with chronic tubulointerstitial nephritis (TIN) as the most frequent histopathological finding in affected cats. CKD is characterized by a progressive loss of renal function, leading to systemic accumulation of waste products, electrolyte imbalances, and impairment of calcium and phosphorus homeostasis. In the most advanced stages, deposition of calcium salts within the renal parenchyma can occur [3,5]. In a study conducted by Mukhopadhyay et al. [42], nephrocalcinosis was evident in 61% of CKD cats, with increased prevalence (81%) in those with hypercalcemia, suggesting a positive correlation with disease progression. Histologically, mineralization most commonly affects the tubular basement membranes and interstitial tissues, particularly within the renal cortex and medulla. Furthermore, toxic exposure to ethylene glycol may result in calcium oxalate crystal deposition within renal tubular lumina, epithelial cells, and the interstitium. The crystals appear as rosettes or prisms that are birefringent under polarized light and are typically accompanied by tubular dilation and necrosis, leading to nephrocalcinosis [29]. In addition, the formation of uroliths in cats is a multifactorial process influenced by dietary composition, metabolic imbalances, genetic predisposition, and urinary tract infections [43], with calcium oxalate (CaOx) as the predominant mineral forming the ureteroliths [44]. Nephroliths may migrate into the ureter and potentially obstruct the urinary flow, leading to hydronephrosis and progressive renal damage [45]. In advanced stages of CKD, dysregulation of calcium and phosphorus homeostasis frequently lead to metastatic calcification of the vascular system and gastric mucosa. Indeed, an impaired renal excretion of phosphate may result in hyperphosphatemia, promoting secondary renal hyperparathyroidism [3,5]. In this context, mineral deposits primarily affect the tunica media followed by the intimal layer, while within the gastrointestinal tract, mineralization involves the mucosa and submucosa, with mineral aggregates often observed along the basement membranes of gastric glands. Uremic gastropathy represents a sequela of advanced CKD in dogs and cats, associated with ulceration in the digestive system, bleeding, and subsequent poor nutritional status [3,5] (Figure 1a,b).

In the context of incidental findings, intratubular mineralization at the renal corticomedullary junction was also described in dogs and cats without clinical signs of renal disease [46]. These deposits are most commonly within the tubular lumina at the corticomedullary junction and appear as discrete foci or as a continuous “band” of basophilic, granular to amorphous material. They are frequently not associated with tubular degeneration, inflammation, or systemic calcium and phosphate imbalance. Similarly, in human medicine, subepithelial calcium phosphate deposits, known as Randall’s plaques, have been described in the renal papilla. These plaques can act as a central focus for calcium oxalate crystal deposition, leading to nephrolithiasis [46]. Since an analogous mechanism has not been established yet in dogs and cats, it is currently unknown whether the incidental corticomedullary mineralization represents the veterinary counterpart of Randall’s plaques.

In addition, mineralization of Bowman’s capsule is often mild and multifocal, and it consists of basophilic deposits along the parietal epithelial basement membrane. This represents a common and nonspecific finding in aging or diseased kidneys, rather than an indicator of systemic metabolic imbalance [3,5]. However, recent clinicopathological studies described that Bowman’s capsule mineralization occurs in a statistically significant higher percentage of proteinuric casts, suggesting a potential association with chronic glomerular injury [47]. These findings raised the possibility that Bowman’s capsule mineralization may serve as a histologic marker of glomerular damage in feline chronic kidney disease. Lastly, calcium carbonate urolithiasis represents a complication in captive mammalian herbivores, often associated with a calcium-rich diet, that potentially may result in urethral or bladder obstruction [48]. In equids, post-necrotic medullary crest calcification may develop as a sequela to papillary necrosis caused by ischemic or nephrotoxic injury, for example, following phenylbutazone administration, representing a species-specific manifestation of dystrophic mineralization within the renal medulla [49].

### 4.3. Skin Calcification

**Figure 2:**
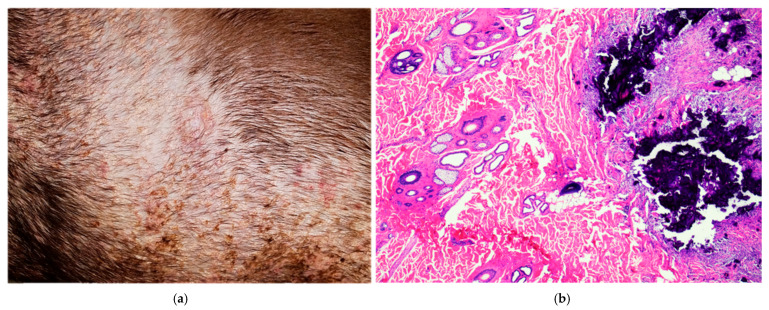
Soft tissue mineralization in domestic species. (**a**) Case L1707997, dog, Pitbull, 10 years. Skin (thorax): marked multifocal to coalescing dermal mineralization with moderate lymphohistiocytic infiltrate. Diagnosis: calcinosis cutis secondary to iatrogenic hypercortisolism and T-cell lymphosarcoma in multiple organs (heart, lung, esophagus, urinary bladder, and temporalis muscle). (**b**) Case L2304357, Dog, Labrador Retriever, 4 years. Skin (dorsum): multifocal dermal lakes of granular basophilic mineral surrounded by fibroplasia and epithelioid macrophages. Diagnosis: calcinosis cutis, secondary to endogenous hypercortisolism.

Calcinosis cutis (CC) is a pathological condition featured by the deposition of inorganic, insoluble mineral salts, mostly calcium phosphate, within the dermis, subcutis, and, less frequently, epidermis [50]. It has been described as nodular aggregates of calcium salts in the tongue and paw pads of dogs, over bony prominences of the limbs in young large breeds of dog, or over the dorsal cervical region or axillae [13] (Figure 2a,b).

CC can be secondary to multiple systemic and localized disorders and is classified into four distinct types: metastatic, iatrogenic, dystrophic, and idiopathic [13]. Metastatic calcification refers to the abnormal deposition of calcium salts in the context of systemic disturbances in calcium and phosphorus metabolism. This form is most frequently observed in dogs with renal insufficiency, but it was also described in the paws of dogs and cats systemically affected by blastomycosis or paecilomycosis [14,51]. The iatrogenic form is secondary to the percutaneous injection of calcium into the skin, which causes localized mineral deposition [52]. Dystrophic calcification is the most frequently encountered form in dogs, and it arises in areas of prior tissue injury followed by iatrogenic hyperglucocorticism or endogenous hyperadrenocorticism. In addition, it may also develop secondarily to systemic infections such as leptospirosis [13]. Several chronic inflammatory dermatoses, such as follicular cysts, foreign body granulomas, interdigital pyoderma, demodicosis, and pilomatrixomas, have been associated with the development of dystrophic calcification [13]. The idiopathic form occurs in young dogs less than one year of age and undergoes spontaneous resolution within the first year of life.

On the other hand, calcinosis circumscripta is a distinct pathological form that appears in pressure points or sites of previous trauma without spontaneous clinical resolution [8]. Microscopically, irregular aggregates of basophilic and granular minerals may elicit a foreign body-type granulomatous inflammatory response with numerous macrophages, multinucleated giant cells, and fibrosis. Lastly, osseous metaplasia, also known as metaplastic ossification of the skin, is an uncommon pathological form featured by the deposition of hydroxyapatite crystals embedded in a proteinaceous matrix. This form of calcinosis circumscripta has been commonly associated with iatrogenic or endogenous hyperglucocorticism in dogs [8].

### 4.4. Muscle Calcification

**Figure 3:**
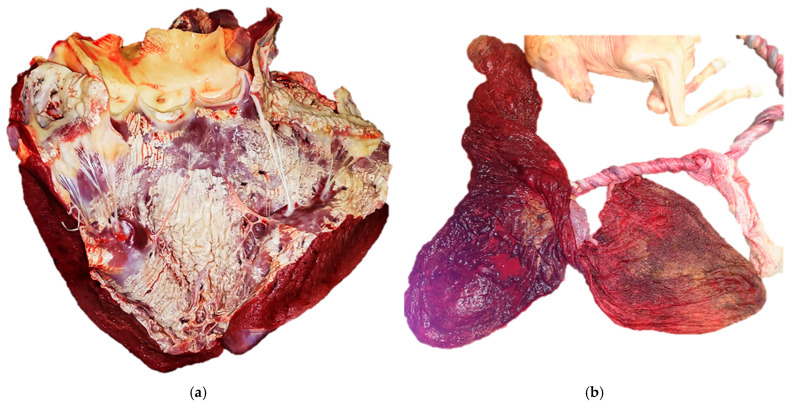
Soft tissue mineralization in domestic species. (**a**) Case L1802778, Horse, Thoroughbred, 2 years. Heart: metastatic alimentary lymphosarcoma with severe diffuse subendocardial mineralization and multifocal mild subepicardial fibrosis. Diagnosis: T-cell lymphosarcoma; (**b**) Case L1800428, Equine Palomino fetus, late-gestation abortion. Placenta and fetus: multifocal chorionic mineralization and thrombosis, hydrocephalus and mild brain edema of the fetus. Diagnosis: abortion associated with excessive umbilical cord length and torsion.

Calcification of skeletal and cardiac muscle represents a form of dystrophic mineralization that is typically secondary to myofiber necrosis. Myofibers have an abundant sarcoplasmic reticulum and following ischemic events or oxidative injury, the increased calcium concentrations trigger the activation of proteolytic enzymes, culminating in myofiber necrosis [53]. On a gross examination, the mineralized muscle appears pale, gritty, or chalky, while microscopically, mineral deposits are basophilic, finely granular to crystalline, and often elicit a granulomatous foreign body response [54]. White muscle disease, also known as Enzootic myopathy, is a nutritional myopathy primarily affecting young ruminants, pigs, and occasionally foals that are deficient of selenium and vitamin E [5]. The heart, diaphragm, and tongue are the sites most affected by segmental myofiber necrosis and dystrophic mineralization [54] (Figure 3a). Similarly, the equine systemic calcinosis described in young Quarter and Paint horses is associated with mineral deposition within both skeletal and cardiac musculature, as well as in the perivascular connective tissues [36,37].

### 4.5. Nervous System Calcification

Mineralization within the nervous system is relatively uncommon but can occur in specific pathological contexts. For example, in horses, siderocalcinosis, which is commonly considered an incidental and age-related finding, is characterized by calcium and iron salt deposition within cerebral arteries without concomitant inflammation and/or glial response [3,5]. However, in a retrospective study conducted by Martinez et al. [38], 31 equine cases of vascular mineralization were retrospectively evaluated. Most frequently affected were venules, arterioles, and/or capillaries within the cerebellar white matter, peduncles, roof nuclei, and internal capsule, with variable degrees of mineral deposition in the tunica media. In most cases, the mineral deposits partially or completely occluded the vascular lumen, and the adjacent parenchyma showed mild lymphocytic infiltration and astrocytosis, with minimal neuronal necrosis. The authors hypothesized that the concentric accumulation of mineral salts within the vascular walls may have caused a progressive narrowing of the vascular lumina, with subsequent ischemic injury, inflammation, and gliosis. It is noteworthy that four horses with neurological signs showed more severe lesions compared to the asymptomatic group, indicating a predisposing role of cerebral siderocalcinosis [38], which could be used as a potential hallmark for lesions.

In aging horses, mineralization may also be observed in association with choroid plexus cholesterol granulomas that are composed of cholesterol clefts, fibrous tissue, and macrophages, while in ethylene glycol toxicosis, the calcium oxalate crystals are commonly within periarteriolar spaces [3,5].

### 4.6. Placenta and Fetal Adnexa Calcification

In veterinary medicine, pathological mineralization of the placenta may arise as a consequence of vascular compromise, ischemia, hemorrhage, or chronic inflammation, all scenarios in which the tissue viability is impaired, inducing dystrophic calcification [5]. For instance, equine chorioallantois is susceptible to mineralization, especially in cases of umbilical cord torsion. Several studies reported that excessively long umbilical cords may increase the risk of torsion and vascular compromise, ischemia, and subsequent mineral deposition within chorionic villi and stroma [55] (Figure 3b). A recent survey on aborted equine fetuses and stillborn foals found that umbilical cord torsion was the most prevalent cause of abortion (≈52%) in the studied cohort [56], followed by pregnancy loss due to a variety of opportunistic bacteria, including bacteria not commonly associated with equine abortion, which accounted for 12% of the cases, and equid alphaherpesvirus (EHV) type 1, which was the cause of pregnancy loss in 8% of the mares. Inflammation and vasculitis secondary to infections represent a common mechanism underlying placental tissue calcification in multiple domestic species. For example, in cattle, a chronic infection by *Ureaplasma diversum* has been associated with necrotizing chorioallantoitis featured by vascular lesions, thrombosis, and mineral deposits [57]. Similar placental mineralization and vascular findings have also been described in abortions or stillbirths associated with pathogens such as *Salmonella* spp., *Brucella* spp., *Coxiella burnetii* spp., and *Leptospira* spp. in ruminants and equines, indicating that inflammatory damage, vascular injury, and subsequent tissue necrosis play a significant role in placenta calcification [58].

Importantly, findings from human and experimental animal placentas demonstrated that calcification is not merely a passive deposition of calcium salts but may represent an active and regulated process involving osteogenic-like pathways. A study demonstrated an increased expression of key osteogenic signaling molecules and structural proteins including osteopontin and osteocalcin in calcified placental tissue, along with higher mineral deposition when compared with non-calcified placentas [59].

Given the diversity of histological structures of the placenta among domestic species, the susceptibility to mineralization may vary; however, the common pathophysiologic mechanism appears to be secondary to vascular injury, inflammation, or ischemia, with calcium salt deposition mostly occurring in vascular walls, stromal tissue, or within necrotic foci. Overall, in humans and domestic animals, placental mineralization should be regarded as a histopathologic indicator of previous vascular damage, inflammatory injury, and/or necrotic processes.

### 4.7. Comparative Pathology of Calcification Across Species

Despite the fact that the molecular mechanisms that drive soft tissue calcification are conserved among different species, there are species-specific metabolic, nutritional, and environmental factors that influence the different lesion distributions and clinical manifestations. In ruminants and horses, dystrophic calcification commonly targets skeletal and cardiac muscle, as in the myodegenerative disorder called white muscle disease, triggered by selenium or vitamin E deficiency [3]. White muscle disease predominantly affects young, rapidly growing animals born from selenium-deficient dams that show clinical signs ranging from acute decompensation to weakness and stiffness [3]. In foals, a similar deficiency can manifest as a peracute, often fatal syndrome or a subacute form with muscular weakness, featured by stunting and markedly elevated muscle enzymes paired with low glutathione peroxidase activity [60]. Vitamin E deficiency in rabbits similarly causes nutritional muscular dystrophy, characterized by degeneration and necrosis of skeletal myofibers and increased plasma creatine phosphokinase and cholesterol concentrations [61].

Dogs and cats commonly exhibit metastatic calcification of renal and gastric tissues secondary to chronic kidney disease or endocrine disorders, resembling human uremic arteriosclerosis [28,62]. Vascular calcification in horses is parallel to human Mönckeberg’s sclerosis [4,20]. On the other hand, idiopathic calcinosis circumscripta in dogs lacks a human correlate, while infection-associated calcifications caused by *Mycobacterium bovis* or *Brucella abortus* mimic the granulomatous and mineralization foci observed in human tuberculosis and brucellosis [63].

## 5. Infectious Agents as Drivers of Pathological Calcification

It has been extensively described that a substantial proportion of mineralized lesions encountered in veterinary pathology occur in association with infectious agents capable of eliciting granulomatous inflammation, caseous necrosis, and persistent infections, warranting recognition as a distinct pathological category. These infectious agents generate a microenvironment that facilitates the precipitation of calcium and phosphate, despite normal calcium and phosphate levels [64].

In this context, bovine tuberculosis is a chronic inflammatory disease caused by *Mycobacterium bovis* that elicits the formation of granulomas as a host-driven response aimed at isolating and containing mycobacterial organisms, while simultaneously mitigating tissue damage [63]. The inflammation mainly affects the lungs and regional lymph nodes, and lesions are classified according to the degree of mineralization, extension of necrosis, and presence or absence of a capsule [65], similarly to human tuberculosis caused by *Mycobacterium tuberculosis*. Furthermore, chronic infections caused by *Brucella* spp. are responsible for necrotizing inflammation, such as orchitis or endometritis in livestock, predisposing the affected tissues to dystrophic calcification in the form of pale and gritty mineralized foci [66]. Given the zoonotic potential for multiple *Brucella* species, these findings are diagnostically significant in veterinary pathology while carrying important implications for zoonotic disease surveillance [66].

On the other hand, protozoal infections, particularly those caused by *Neospora caninum*, are associated worldwide with reproductive loss in cattle and neuromuscular disease in companion animals [67]. In ruminants, scattered pale to dark tan foci in the brain, spinal cord, heart, and skeletal muscle are indicative of necrosis and inflammation, while in young dogs neosporosis is featured by widespread myofiber necrosis with fine granular calcification and pale streaks in the skeletal muscle [67].

Similarly, helminthic infections can also lead to calcified lesions. For example, *Taenia hydatigena* is a ubiquitous tapeworm described in definitive hosts, such as dogs and other carnivores and ruminants, pigs, and wild boars that act as intermediate hosts. During the migratory phase of the *T. hydatigena*, common findings in pigs include hepatic hemorrhage and necrosis, with frequent peripheral calcification [68].

Dimorphic fungi such as *Histoplasma capsulatum*, *Blastomyces dermatitidis*, and *Coccidioides* spp. are important etiologic agents of systemic mycoses in veterinary medicine, and they elicit chronic granulomatous or pyogranulomatous inflammation in multiple tissues, including the lungs, lymph nodes, skin, eyes, brain, and bone [3]. In prolonged infections, granulomas may also undergo dystrophic mineralization due to the long-standing tissue necrosis and inflammation. For example, in histoplasmosis, calcification affects the pulmonary granulomas [42], while in canine blastomycosis, thoracic lymph nodes may contain firm and mineralized granulomas [5]. The identification of soft tissue calcification in the context of infectious disease has significant diagnostic relevance. Indeed, the presence of mineral deposits within granulomatous or necrotizing inflammatory foci should prompt the implementation of special stains, such as Ziehl Neelsen staining for acid-fast bacteria, immunohistochemistry for protozoal antigens, or Gomori methenamine silver staining for fungal pathogens.

Multiple pathogens responsible for soft tissue calcification in animals are zoonotic, including *Mycobacterium bovis*, *Brucella* spp., *Toxoplasma gondii*, and *Echinococcus granulosus*, and misinterpretation of such lesions may potentially delay an appropriate diagnosis, zoonotic risk assessment, and implementation of public health measures [5]. This underscores the translational role of veterinary medicine surveillance from a public health standpoint. In addition, the identification of calcified lesions in wildlife hosts represents valuable surveillance markers, enhancing monitoring on an ecosystem level and within a One Health perspective.

## 6. Future Research Directions

Growing evidence supports the hypothesis that calcium deposition may be triggered by active and regulated biochemical processes in addition to being a passive precipitation of calcium salts. In this regard, several proteins involved in osteogenesis were detected in calcified arterial walls, while VSMCs were found to be able to differentiate into an osteogenic and chondrogenic phenotype [4]. Despite significant advances in the characterization of mineralization processes, multiple aspects of ectopic calcification still require further investigation. Indeed, further studies are needed to define the signaling pathways triggering calcification in various tissues and to elucidate the role of calcification promoters and inhibitors across species [6,20]. The causes of idiopathic calcification, including calcinosis circumscripta, remain undetermined and future studies are required to better understand genetic, metabolic, and microenvironmental contributing factors. Similarly, the significance of subclinical mineralization across species remains poorly understood, as in the case of arterial medial calcification in young racing horses. Moreover, the growing integration of high-throughput technologies, such as transcriptomics and proteomics, into veterinary diagnostic workflows holds significant promises for elucidating pathogenesis and for the discovery of novel biomarkers of ectopic mineralization.

Notably, insights from comparative pathology have demonstrated that soft tissue calcification in animals resembles the molecular mechanisms of physiological bone formation, underscoring the translational value of veterinary pathology in advancing the knowledge of calcification-related disorders.

## 7. Summary and Conclusions

Tissue calcification represents a significant and heterogeneous pathological process in veterinary medicine, characterized by the deposition of insoluble calcium salts in soft tissues. It is commonly categorized into dystrophic, metastatic, idiopathic, and iatrogenic, based on distinct etiological and pathological mechanisms. Dystrophic calcification typically arises from areas of necrosis or injury, whereas metastatic calcification results from a systemic mineral imbalance, such as hypercalcemia or hyperphosphatemia. Idiopathic forms occur in the absence of an identifiable cause, while iatrogenic cases result from the administration of or exposure to exogenous calcium. Calcification is diagnosed in a broad range of tissues and species, including the kidney and lungs in cats with chronic kidney disease, arteries in horses, and skin in calcinosis cutis in dogs. Therefore, an accurate diagnosis requires an integration of anamnesis, clinical history, and clinicopathological values with a detailed gross and microscopic examination. In addition, special stains, including von Kossa or Alizarin Red S staining, are essential to distinguish calcification from other extracellular deposits. In veterinary pathology there are numerous advantages deriving from the possibility to investigate naturally occurring calcification across multiple animal tissue specimens collected at various stages of disease progression. This longitudinal approach, which facilitates a more comprehensive understanding of lesion development and progression, is often not feasible in human medicine due to limitations in tissue sampling. In conclusion, tissue calcification in animals may provide valuable insights into disease mechanisms and diagnostic challenges, with comparative pathology serving as a powerful tool to enhance our understanding of these processes. This knowledge not only protects animal health but also supports translational applications relevant to human medicine.

## Figures and Tables

**Table 1:** Types of pathological calcification in veterinary medicine and organ- and species-specific examples.

Category	Pathogenesis	Organ/System	Species-Specific Examples
Dystrophic	Injured or necrotic tissue following inflammation, degeneration, or thrombosis Necrotic tissue secondary to ischemic or toxic injury Serum calcium and phosphate concentrations within normal range	Muscle	“White muscle disease” in young ruminants, pigs, and foals (vitamin E/selenium deficiency) Equine systemic calcinosis with skeletal and cardiac muscle mineralization
		Skin	Calcinosis cutis in dogs with hyperadrenocorticism (endogenous or iatrogenic) Calcinosis circumscripta at pressure points or trauma sites
		Blood vessels	Arterial calcification in horses following repeated hemodynamic stress Arterial medial calcification in rabbits, guinea pigs, and rats
		Urinary system	Tubular and interstitial mineralization in chronic infections or toxic injury (ethylene glycol toxicity) Post-necrotic medullary crest calcification in horses
Metastatic	Secondary to systemic imbalance of calcium and phosphate levels, such as hypercalcemia and/or hyperphosphatemia	Kidney	Chronic kidney disease Vitamin D intoxication Primary or secondary hyperparathyroidism Paraneoplastic syndromes
		Gastrointestinal	Uremic gastropathy secondary to CKD
		Blood vessels	Arterial mineralization secondary to CKD Arterial mineralization secondary to vitamin D toxicosis
		Skin	Renal insufficiency Systemic fungal infections (*Blastomyces*, *Paecilomyces*)
Idiopathic	In the absence of tissue damage or abnormality in the metabolism of calcium or phosphorus	Skin	In young dogs without history of glucocorticoid exposure
		Soft tissue	Nodular aggregates in dog’s tongue and paw pads
Iatrogenic	Direct exposure to exogenous calcium-containing compounds leading to local injury Calcium-rich diet	Skin	Localized mineral deposition following percutaneous injection of calcium
		Urinary system	Calcium carbonate urolithiasis in captive mammalian herbivores
Metaplastic (Heterotopic ossification)	Formation of mature bone or cartilage in chronic lesions of soft tissues calcifications	Muscle/connective tissue/arteries	Bone formation in paravertebral muscles causing lameness in dogs Osseous metaplasia secondary to a salivary mucocele Lamellar bone formation in the aortic valve of a horse
Infection-associated calcification	Persistent and/or chronic infections causing necrosis and inflammation Often zoonotic relevance	Lung/regional lymph nodes	Mineralized granulomas in bovine tuberculosis
		Reproductive tract	Calcification in endometritis and orchitis caused by Brucella species Placental chorion mineralization in several bacterial infections (*Brucella* spp., *Leptospira* spp., *Coxiella burnetii*, *Salmonella* spp., and *Ureaplasma* spp.)

## Data Availability

No new data were created or analyzed in this study. Data sharing is not applicable to this article.
